# Clinical and genetic validity of quantitative bipolarity

**DOI:** 10.1038/s41398-019-0561-z

**Published:** 2019-09-16

**Authors:** Heather A. Bruce, Peter Kochunov, Braxton Mitchell, Kevin A. Strauss, Seth A. Ament, Laura M. Rowland, Xiaoming Du, Feven Fisseha, Thangavelu Kavita, Joshua Chiappelli, Krista Wisner, Hemalatha Sampath, Shuo Chen, Mark D. Kvarta, Chamindi Seneviratne, Teodor T. Postolache, Alfredo Bellon, Francis J. McMahon, Alan Shuldiner, L. Elliot Hong

**Affiliations:** 1 Maryland Psychiatric Research Center, Department of Psychiatry, University of Maryland School of Medicine, Baltimore, MD 21228 USA; 2 Department of Medicine, University of Maryland School of Medicine, Baltimore, MD 21228 USA; 3 Clinic for Special Children, Strasburg, PA 17579 USA; 4 Department of Psychiatry, University of Maryland School of Medicine, Baltimore, MD 21228 USA; 5 Hershey Medical Center, Department of Psychiatry, Penn State University School of Medicine, Hershey, PA 17033 USA; 6 Human Genetics Branch, National Institute of Mental Health Intramural Research Program, Bethesda, MD 20892 USA

**Keywords:** Clinical genetics, Medical genetics

## Abstract

Research has yet to provide a comprehensive understanding of the genetic basis of bipolar disorder (BP). In genetic studies, defining the phenotype by diagnosis may miss risk-allele carriers without BP. The authors aimed to test whether quantitatively detected subclinical symptoms of bipolarity identifies a heritable trait that infers risk for BP. The Quantitative Bipolarity Scale (QBS) was administered to 310 Old Order Amish or Mennonite individuals from multigenerational pedigrees; 110 individuals had psychiatric diagnoses (20 BP, 61 major depressive disorders (MDD), 3 psychotic disorders, 26 other psychiatric disorders). Familial aggregation of QBS was calculated using the variance components method to derive heritability and shared household effects. The QBS score was significantly higher in BP subjects (31.5 ± 3.6) compared to MDD (16.7 ± 2.0), other psychiatric diagnoses (7.0 ± 1.9), and no psychiatric diagnosis (6.0 ± 0.65) (all *p* < 0.001). QBS in the whole sample was significantly heritable (h^2^ = 0.46 ± 0.15, *p* < 0.001) while the variance attributed to the shared household effect was not significant (*p* = 0.073). When subjects with psychiatric illness were removed, the QBS heritability was similar (h^2^ = 0.59 ± 0.18, *p* < 0.001). These findings suggest that quantitative bipolarity as measured by QBS can separate BP from other psychiatric illnesses yet is significantly heritable with and without BP included in the pedigrees suggesting that the quantitative bipolarity describes a continuous heritable trait that is not driven by a discrete psychiatric diagnosis. Bipolarity trait assessment may be used to supplement the diagnosis of BP in future genetic studies and could be especially useful for capturing subclinical genetic contributions to a BP phenotype.

## Introduction

Bipolar disorder (BP) affects about 1% of the population, causing significant disability worldwide. BP is considered a highly heritable condition with an estimated 40–80% heritability^1–3^. Case-control genome-wide association studies (GWAS) are beginning to illuminate the genetic risk for this complex polygenic disorder and have identified a number of loci^4–6^ though findings have been difficult to replicate. Most genetic studies of BP define the study group by diagnosis. While this definition is important for clinical care of BP patients, whether this is the correct phenotype to use to search for genes conferring risks for BP is not clear. Genetically susceptible individuals without BP expression may be missed or even erroneously grouped into the control groups. Our hypothesis is that bipolarity is not only expressed in BP but may also be a heritable subclinical trait that is present even in non-bipolar individuals, yet much more severe in BP and as such separates BP from other psychiatric diagnoses.

The Bipolar Spectrum Diagnostic Scale (BSDS) is a self-report scale designed to screen for bipolar spectrum disorders^7^. BSDS was shown to have a sensitivity of 0.75 and a specificity of 0.93 for BP in an outpatient clinic population^7^, a range that was generally supported by other studies^8–11^. These data support the clinical validity of BSDS to quantify clinical and subclinical bipolarity symptoms. The original BSDS does not include all DSM-5 BP symptoms. We adopted much of the BSDS content and format but modified items to cover symptoms in the DSM-5 bipolar I criteria, and also implemented a new severity rating for each item, henceforth called the Quantitative Bipolarity Scale (QBS). To our knowledge the heritability of BSDS or other quantitative bipolarity tools, such as the Mood Disorders Questionnaire (MDQ)^12^, has never been evaluated. Although QBS is designed to be consistent with DSM-5, it is completed by patients, allowing independent evaluations of its validity using clinician assessed DSM-5 diagnoses.

The ideal sample to test whether a quantitative bipolarity measurement is a heritable endophenotype would be a population with BP and with a family structure which enables estimation of genetic vs non-genetic effects. Pioneered by Egeland, there has been a long history of studying the genetics of BP through large pedigrees in the Amish/Mennonite population^3,13^. The Old Order Amish and Old Order Mennonite (OOA/M) population is a founder population whose large families and genealogical record keeping make the population a powerful resource for genetic and heritability analyses even in modest sample sizes. The prevalence of BP in the OOA/M appears similar to that in the general population^14^ though some pedigrees carry a heavier burden of mood disorders^3,15^. In addition, they share similar rural upbringing and school education. The high environmental homogeneity reduces between-subject variations of uncontrolled developmental and environmental factors theoretically yielding more precise estimates of genetic contributions. The low incidence of substance use disorders decreases another confounding factor commonly present in BP patients in the general population. Therefore, this study aims to develop a potentially novel phenotyping alternative to supplement traditional diagnosis-based genetic research in BP by taking advantage of the large family structures in the OOA/M, to test the hypothesis that QBS provides a heritable quantitative trait that separates BP from other psychiatric diagnoses.

## Methods and materials

### Subjects

**Fig. 1:**
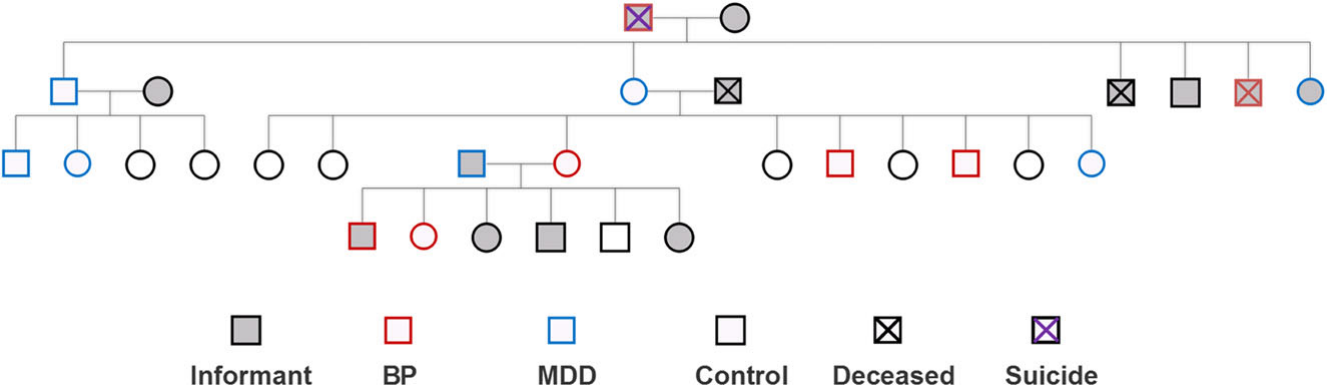
An example pedigree. Individuals whose diagnoses were estimated based on informant reports did not participate in the study. Some members and birth orders were removed or altered to mask the family identity. MDD major depressive disorder, BP bipolar disorder

The study included 310 members of OOA/M families [134 male, 176 female, age (42.3 ± 18.7, mean ± s.d.)] from 119 nuclear families in Pennsylvania and Maryland. The sample included 173 sibling (sibship size ranged from 2 to 9), 25 spouse pair, and 185 parent-child pair relationships. Sixty nuclear families had one individual participating (although they are related to other members in the extended pedigrees at second or third degree levels), 24 families had two participants, 35 families had three or more participants. As this is a founder population and marriages are kept within the community, most families are connected using genealogical records maintained by the OOA^16^ and the OOM communities^17^ and digitalized in the NIH Anabaptist Genealogy Database (AGDB)^18^. The genealogical data were converted to the pedigree format by the SOLAR-Eclipse software (http://www.nitrc.org/projects/se_linux). Exclusion criteria included major medical and neurological conditions and substance abuse in the past year. Recruitment was based on the Research Domain Criteria (RDoC) strategy^19^ and included all Axis-I psychiatric illnesses, starting with identifying families with at least two cases of any psychiatric illnesses and then followed by recruiting members from the same household regardless of diagnosis. Families without psychiatric illnesses were also recruited. Note that this traditional definition of case and control families is a relative term in a population isolate where families are interrelated. For this study individuals without psychiatric illness were labeled as controls irrespective of their family status. Figure 1 gives an example of one pedigree. Only individuals that were directly interviewed were included in the analysis.

The data included 110 individuals with a lifetime diagnosis of psychiatric disorders: bipolar disorders (*n* = 20, including 19 bipolar I and 1 bipolar II), major depressive disorder (MDD) (*n* = 61), schizophrenia spectrum disorders (*n* = 3), and other psychiatric disorders (*n* = 26, including: other specified depressive disorder^5^, persistent depressive disorder^3^, premenstrual dysphoric disorder^2^, adjustment disorder^1^, attention deficit hyperactivity disorder^1^, substance use disorder^3^, social anxiety disorder^3^, panic disorder^3^, obsessive compulsive disorder^2^, generalized anxiety disorder^1^, other specified anxiety disorder^2^) and 200 individuals with no lifetime psychiatric disorders. The Schedule for Clinical Interview for DSM-5 (SCID-5) was used to determine diagnoses by trained clinicians. Each SCID interview was reviewed in consensus meetings. For SCID inter-rater reliability, a research team from another study interviewed 17 of the current OOA/M participants^15^. Diagnoses for 15 of 17 subjects were in agreement while 2 had minor differences (kappa = 0.87), supporting the reliability of the SCID in this population. All study participants gave written informed consent as approved by the University of Maryland IRB.

### Quantitative bipolarity rating scale (QBS)

The original BSDS contains 19 items and a summary item^7^ in a format designed to extract the polarity of mood by self-report. We revised the BSDS to reflect DSM-5 criteria for bipolar I disorder. For example, the DSM symptoms “inflated self-esteem or grandiosity” and “decreased need for sleep” were not represented in the original BSDS. In the revision, we added items addressing those symptoms as well as items addressing racing thoughts and distractibility. The original BSDS had one item “may be more talkative, outgoing, or sexual” which we separated into three items as they are represented by more than one symptom criterion in DSM-5. Also an item regarding increased substance use during periods of elevated mood was removed as this is not a specific criterion for BP in DSM-5. Furthermore, as we aimed to develop a quantitative phenotype, a severity scale was implemented for each item. Subjects were asked to rate each item from 0 to 3 (0 for “this description doesn’t really describe me at all”, 1 for “this description fits me to some degree but not in most respects”, 2 for “this description fits me fairly well”, and 3 for “this description fits me very well or almost perfectly.”)

The QBS analysis was based on the summed values from all individual items as well as a item asking how much the scale as a whole describes the individual. Additional analysis was performed on predefined subscale scores. Items 3 through 8 refer to depressive symptoms. Items 11 through 24 refer to manic symptoms. Items 1, 2, 9, and 10 refer to mood fluctuation symptoms. Hereafter these respective sums are referred to as depression subscore, mania subscore, and mood fluctuation subscore. The Quantitative Bipolarity Scale (QBS) is available online (www.mdbrain.org/QBS_instructions_and_scale.pdf).

### Statistical analysis

Linear mixed-effects model fit by maximum likelihood estimation was used to compare QBS scores across diagnostic groups, where age and sex were fixed effects and familial relationships were random effects. This procedure was repeated for QBS subscores. Receiver Operating Characteristic (ROC) curve was performed to evaluate sensitivity and specificity of QBS. Cutoff was based on the Youden-Index to determine the point for which sensitivity plus specificity is maximal^20^.

Heritability estimates were obtained using the variance components method as implemented in the SOLAR-Eclipse software package (http://www.nitrc.org/projects/se_linux). Heritability (h^2^) is defined as the proportion of the total phenotypic variance that is explained by additive genetic factors in related individuals. The variance parameters are estimated by comparing the observed phenotypic covariance matrix with the covariance matrix predicted by kinship. Inverse Gaussian transformation was applied to ensure normality of the measurements. Household effects were simultaneously estimated for shared environmental effects. Siblings were grouped in the same household. Significance of the heritability is tested by comparing the likelihood of the model in which additive genetic factors is constrained to zero with that of a model in which additive genetic factors is estimated. Twice the difference between the log_e_ likelihoods of these models yields a test statistic^21^. Age and sex were used as covariates when calculating the heritability of QBS scores. The familiality of QBS score was further evaluated by comparing family members (first or second degree relatives) of individuals with high QBS score (defined by QBS score cutoff > 23, which is the mean plus one SD of the entire group) to the remaining individuals on QBS scores.

We further estimated the extent to which QBS score and BP diagnosis (coded as 1 for bipolar diagnosis and 0 for controls) were explained by shared genetic factors. The genetic correlation (ρG) of the two traits is modeled as a linear function of kinship coefficients that express relatedness among all pairs of individuals in the pedigree; the phenotypic variance–covariance matrix and its additive genetic and random environmental components are then obtained. The significance of the components are then estimated directly by the likelihood ratio test^22,23^. If ρG is significantly different from zero then a significant proportion of the traits’ covariance is considered to be influenced by shared genetic factors^22^. Inverse Gaussian transformation was applied to achieve normality of the QBS measure.

To further explore whether there are latent structures of QBS not captured by the predefined depression, mania, and mood fluctuation subscales, factor analysis was performed using the whole sample. Principal axis factor analyses were performed to identify the latent constructs in the data using oblique (promax) rotations. The optimal solution was based on a combination of common factor solution eigenvalues > 1.0, factor structure using loadings > 0.35, and percent of variance explained.

## Results

### Clinical validity

Age and sex ratio did not differ significantly across diagnostic groups (Table 1). The QBS score was significantly higher in the BP group compared to all other groups [F_3,305_ = 30.3, *p* < 0.001] (Table 1). Post-hoc tests showed that there was a significant difference between the QBS score in subjects with BP (31.5 ± 3.6, mean ± s.e.) compared to MDD (16.7 ± 2.0), other psychiatric diagnosis (7.0 ± 1.9), and no psychiatric diagnosis (6.0 ± 0.65) (all *p* < 0.001). The difference was not significant when comparing BP and psychotic disorders (14.7 ± 1.9, *p* = 0.12) although this may be due to the limited number of individuals with psychotic disorder in the sample (Table 1).

**Table 1:** Sample Demographics, QBS (quantitative bipolarity scale) score and QBS subscores across diagnostic groups

	Bipolar disorder	Major depressive disorder	Psychotic disorder	Other psychiatric illness	Control	Test statistic (F or x^2^)	*P-*value
N	20	61	3	26	200		
Gender (Male:female)	12:8	21:40	1:2	12:14	88:112	1.2	0.31
Age	50.3 ± 3.1	42.4 ± 2.1	41 ± 16	44.4 ± 3.3	41.1 ± 1.4	4.5	0.35
QBS score	31.5 ± 3.6*	16.7 ± 2.0	14.7 ± 4.9	7.0 ± 1.9	6.0 ± 0.6	30.6	2 × 10^−21^
Mood fluctuation subscore	5.9 ± 0.7*	3.6 ± 0.4	3 ± 1	2.0 ± 0.6	1.5 ± 0.2	18.5	1 × 10^−13^
Depression subscore	6.4 ± 1.1	5.0 ± 0.5	3.3 ± 0.9	2.0 ± .06	1.2 ± 0.1	30.6	2 × 10^−21^
Mania subscore	17.7 ± 2.0*	7.2 ± 1.2	7.3 ± 3.2	2.4 ± 0.8	2.9 ± 0.4	28.1	7 × 10^−20^

Data are recorded as mean ± standard error. Asterisk indicates measure with significant difference (*p* < 0.5) between bipolar disorder and major depression

**Fig. 2:**
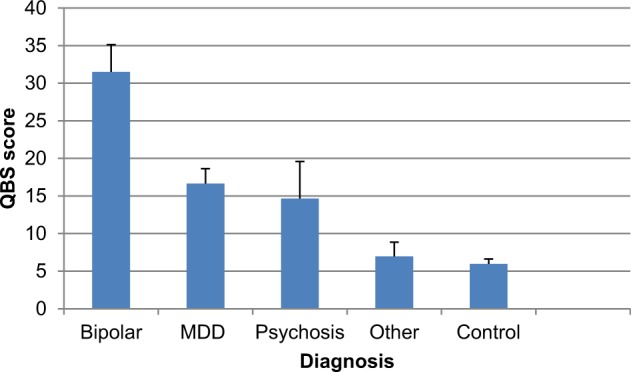
QBS (quantitative bipolarity scale) score across diagnostic groups. Mean QBS score is shown for each of five diagnostic groups[bipolar disorder (*n* = 20), major depressive disorder (MDD) (*n* = 61), Psychotic Disorder (*n* = 3), other psychiatric diagnosis (*n* = 26), controls (*n* = 200). Error bars represent standard error

The QBS score of bipolar patients was approximately double that of MDD, and individuals with MDD showed a two to three fold higher mean QBS score compared to individuals with other psychiatric illnesses. Controls showed the lowest mean QBS score (Fig. 2). These characteristics support the specificity and sensitivity of this scale. We further compared patients with currently symptomatic BP (31.9 ± 4.4; *n* = 15) to patients who have lifetime BP but are currently in full remission (30.2 ± 7.0; *n* = 5), and found that their QBS scores were not significantly different (t = 0.20, *p* = 0.8). This suggests that QBS captures the longitudinal trait aspect of bipolarity symptoms. There was no correlation between age and QBS score (r = −0.09, *p* = 0.11).

**Fig. 3:**
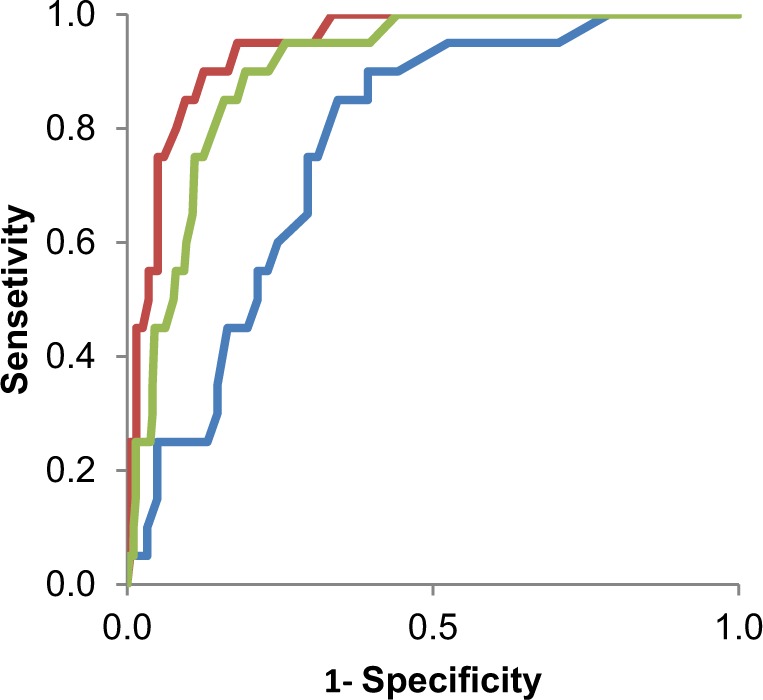
Receiver Operating Characteristic (ROC) Curves. Lines show the ROC curves for the quantitative bipolarity scale distinguishing bipolar disorder vs controls (red), non-bipolar psychiatric illness (green), and major depressive disorder (blue)

The validity of QBS in terms of specificity and sensitivity was formally investigated with a ROC curve analysis. For BP vs controls, sensitivity and specificity was 0.90 and 0.88; for BP vs. all other psychiatric diagnoses, sensitivity and specificity was 0.90 and 0.81; for BP vs MDD sensitivity and specificity was 0.90 and 0.61 (Fig. 3 and Table 2).

**Table 2:** Characteristics for each comparison in Fig. 3

Comparison Group	Sensitivity	Specificity	AUC	SE	95% C.I.	*p*-value
Controls	0.9	0.88	0.94	0.02	0.91–0.98	6 × 10^−11^
Other psychiatric illness	0.9	0.81	0.90	0.02	0.86–0.95	1 × 10^−9^
Major depressive disorder	0.9	0.61	0.77	0.05	0.67–0.88	3 × 10^−4^

AUC is area under the curve, SE is standard error. All comparisons used a cutoff score of 16 based on the Youden index

In terms of the subscales, mood fluctuation, depression, and mania subscores were all significantly different across the five diagnostic groups (Table 1). The key post-hoc tests were comparisons between BP and MDD. This analysis revealed that the mood fluctuation and mania subscores were significantly different between the two groups (*p* = 0.006 and *p* = 6 × 10^−9^ respectively), but the depression subscore was not (*p* = 0.34). This supports the validity of QBS for identifying the converging symptoms (depression) and diverging symptoms (mania and mood fluctuation) characterizing these two major mood disorders.

Factor analyses identified two factors that together accounted for 60% of the variance in the data. All depression items and mood fluctuation items loaded onto factor 1 and all manic items loaded onto factor 2.

### Genetic validity as measured by familial aggregation

Consistent with the literature, a diagnosis of BP was highly heritable in this sample (h^2^ = 0.71 ± 0.45, *p* = 0.03). The QBS score also was significantly heritable (h^2^ = 0.46 ± 0.15, *p* = 4 × 10^−4^) (Table 3). Repeating the QBS analysis with adjustment for shared environment (household effects), the heritability remained significant (h^2^ = 0.38 ± 0.17, *p* = 0.008) and the proportion of phenotypic variance attributable to household effects was not significant (0.12 ± 0.09, *p* = 0.07) (Table 3). One possibility is that the significant heritability of QBS was driven by the presence of BP cases. However QBS heritability remained significant even when removing all bipolar cases (h^2^ = 0.55 ± 0.15, *p* = 4 × 10^−5^). Lastly, we removed all psychiatric illnesses and found that the heritability of the QBS score remained similarly significant (h^2^ = 0.59 ± 0.18, *p* = 4 × 10^−4^) while the phenotypic variance from shared environment in controls was zero (Table 3). It is unclear why the heritability of QBS in controls was greater than that in the subsample without BP, which was greater than that in the whole sample; however, standard methods^24^ indicate that these were not statistically significant differences.

**Table 3:** Heritability of QBS (quantitative bipolarity scale) score

	Whole sample (*n* = 310)	Whole sample without bipolar (*n* = 190)	Non-psychiatric control subjects (*n* = 200)
Unadjusted			
h^2^	0.46(0.15)	0.55(0.15)	0.59(0.18)
p	4 × 10^−4^	4 × 10^−5^	2 × 10^−4^
Age and sex effect (R^2^)	0.01	0.03	0.05
Adjusted			
h^2^	0.38(0.17)	0.50(0.17)	0.59(0.18)
p(h^2^)	0.008	0.002	2 × 10^−4^
Household	0.12(0.09)	0.05(0.08)	0
p(Household)	0.07	0.25	–
Age and sex effect (R^2^)	0.01	0.03	0.05

Additive heritability estimates [h^2^(SE)] are shown with and without adjustment for shared environment. Household is the proportion of the phenotypic variance attributed to shared environment (household effects). Both models included age and sex as covariates. R^2^ is the phenotypic variance explained by the covariates age and sex (none was significant)

An alternative analysis of familiality showed that individuals with a first or second degree relative with a high QBS score had a significantly higher QBS score (7.9 ± 0.85, *n* = 82) than individuals without such relatives (5.2 ± 0.47, *n* = 192) [t(272) = 2.9, *p* = 0.01)].

In further support of genetic validity, genetic correlation analyses demonstrated significant shared genetic variance between BP and QBS score (ρG = 0.55, *p* = 0.04).

Exploring the heritability of the QBS subscores, we found that the mood fluctuation, depression, and mania subscores all had significant heritability (h^2^ = 0.44 ± 0.15, *p* = 4 × 10^−4^; h^2^ = 0.34 ± 0.15, *p* = 0.007; h^2^ = 0.44 ± 0.15, *p* = 7 × 10^−4^, respectively). In addition, all QBS subscores remained significantly heritable when the analysis was performed on controls only (h^2^ = 0.42 ± 0.18, *p* = 0.006; h^2^ = 0.49 ± 0.20, *p* = 0.003; h^2^ = 0.43 ± 0.16, *p* = 0.002, respectively). These subscore findings suggest that the significant heritability of QBS is not driven by any one facet of the scale.

## Discussion

We investigated whether bipolarity quantification can capture the genetic risk for BP beyond that obtained by categorical diagnoses. We found that a concise bipolarity quantification tool using a self-report format that covers symptoms in the current DSM-5 for BP is significantly heritable in pedigree samples from the OOA/M population, interestingly even in individuals without BP or psychiatric diagnosis.

BP is a highly heritable condition. GWAS of BP conducted outside the Amish have identified ~30 significant loci though many have not been replicated^4,25,26^, which indicates that the strong heritability of BP may involve polygenicity and heterogeneity. The genetic loci that may be associated with QBS are yet to be determined. BP has been associated with 11q12.2 region and also a number of single nucleotide polymorphisms including those in the *ODZ4* (*TENM4*,encoding teneurin transmembrane protein 4), *MAD1L1* (encoding mitotic arrest deficient-like 1), and *TRANK1* (encoding tetratricopeptide repeat and ankyrin repeat containing 1) genes^6^. It would be interesting to determine whether using the QBS phenotype can replicate some of these loci associated with BP or whether QBS identifies additional candidate loci. In addition to GWAS studies, more recently whole-genome or -exome sequencing has been used in the search for BP related genes. As rare alleles can be enriched in founder populations, with better characterized phenotypes, the OOA/M may provide an important cohort for discovering risk variants for BP^3,15^.

Use of diagnosis as the primary phenotype in the search for genes conferring risk for BP has often gone unquestioned though some have investigated temperamental traits as endophenotypes for bipolar disorder^27–29^. It is known that BP is prone to over- or under-diagnosis^30^, which has led to the development of tools to screen for BP such as the MDQ and BSDS. A quantitative trait measuring bipolarity may be more informative than diagnosis for genetic studies^31^. Furthermore, as the onset of BP can occur later in age, future patients can be erroneously included as controls or with diagnoses of MDD that may convert to BP later, an issue particularly problematic in pedigree-based genetic search where candidate gene identification has been largely dependent upon comparing case vs non-case status within pedigrees. A tool that can identify subclinical bipolar symptoms and their genetic influence may address this important concern. The bivariate genetic correlation analysis here showed a significantly shared genetic correlation between QBS and BP, supporting that this quantitative bipolarity is at least partially tagging the genetic risk of BP.

This measure of bipolarity scored much higher in BP and clearly separated BP from other psychiatric illnesses. However, the detection of subthreshold bipolarity may also be of value. Subthreshold bipolarity has been extensively studied^32,33^ and may indicate undiagnosed bipolar features in the general population or patients with MDD^34^ and may even predict the conversion of unipolar depression to BP^35^. This view is consistent with our findings in which patients with MDD have a three times higher total bipolarity score, as well as higher mania, mood fluctuation, and depression subscores, compared to non-psychiatric controls.

One of the limitations of this study is that the QBS findings were not evaluated in the general population. However, although OOA/M are culturally and environmentally separated from other Caucasians of European descent in North America, there is evidence that the clinical presentation of mood disorders is similar^36^. The original version of the BSDS has been applied to several populations leading to a range of values for sensitivity (0.70–0.90) and specificity (0.51–0.93) of bipolar vs non-bipolar patients depending on the cutoff score used^7–11^. Using the QBS, the sensitivity and specificity were comparable in OOA/M, which supports the generalizability of our findings.

Furthermore, research indicates that genetic findings in the OOA/M may be highly applicable to the larger population. For example, the contactin-associated protein-2 (CNTNAP2) gene, first associated with autism in the OOA/M^37^, has since been replicated in multiple studies in the general population^38,39^. Similarly, genetic studies of non-psychiatric traits have identified associations and biological mechanisms that are replicable in the general population^40,41^.

One of the novel findings of this study in the OOA/M cohort is that the heritability of QBS was substantial even in subsamples without psychiatric diagnosis, suggesting that the range of subtle bipolarity symptoms assessed by QBS is not specific to mental illness but rather that QBS may be indexing a heritable trait characterized along a continuous dimension from subclinical to overtly clinical symptoms. However, controls in families of a founder population with many cases of BP, may have inflated risk as they are genetically more closely related than discreet families in cohorts from the general population. Therefore, this particular conclusion must be re-tested by applying QBS to family or twin samples from the general population. However, even if this caveat of inflated risk is true, it may actually further support QBS as a marker of genetic risk for BP. In addition, a OOA/M cohort with more densely sampled nuclear families than ours may be helpful in a replication study.

To the best of our knowledge, there has not been a tool designed to quantitatively assess mania vs depression vs mood fluctuation and then used to assess their relative heritability. The three quantitative subscales for mood fluctuation, depression, and mania within QBS were each found to be significantly heritable. Our definitions of the subscores were based on the clinical description of each item. Factor analysis confirmed the clear distinction between manic and depression items. It is unclear why the depression and the mood fluctuation items clustered together. We speculate that it may reflect fluctuations between normal and depressed moods occurring more commonly than fluctuations between normal and manic moods. Factor analysis of QBS in a larger sample of BP may allow a clearer distinction.

Another notable limitation of this study is the lack of QBS administration over time, thus we did not address reliability. Our study and other studies using the BSDS address validity with measures of sensitivity and specificity, however, we do not know of any studies that address reliability. Furthermore we did not evaluate the predictive power of QBS for risk of conversion to BP, which we plan to address in subsequent studies. Some individuals in our study classified as controls were below the age of maximum risk for BP and may go on to develop BP. However this may also be a strength of the QBS approach because a main purpose of this study is to investigate quantitative bipolarity with and without BP. Another limitation of this study is the low number of individuals with a schizophrenia spectrum disorder (1% of the current sample, which is epidemiologically similar to the rate in the general population), given the data supporting a genetic overlap between schizophrenia and BP^42^. An additional limitation is that only about 6% of the current sample have BP but their QBS scores are by definition higher and predominate in the distribution of the overall sample, which is a limitation of the current sample. Future studies recruiting BP based samples are needed to further validate the questionnaire.

Heritability is only the first step of genetic validity; whether QBS would assist in the search for genes conferring risk of developing BP remains to be seen. This study suggests that quantitative bipolarity as measured by the concise self-administered QBS task may be a useful phenotype in supplementing the diagnosis phenotype in BP genetic studies.

## References

[1] Bertelsen A, Harvald B, Hauge M (1977). A Danish twin study of manic-depressive disorders. Br. J. Psychiatry.: J. Ment. Sci..

[2] Craddock N, Sklar P (2013). Genetics of bipolar disorder. Lancet (Lond., Engl.)..

[3] Georgi B (2014). Genomic view of bipolar disorder revealed by whole genome sequencing in a genetic isolate. PLoS Genet..

[4] Psychiatric GWAS Consortium Bipolar Disorder Working Group. (2011). Large-scale genome-wide association analysis of bipolar disorder identifies a new susceptibility locus near ODZ4. Nat. Genet..

[5] Baum AE (2008). A genome-wide association study implicates diacylglycerol kinase eta (DGKH) and several other genes in the etiology of bipolar disorder. Mol. Psychiatry.

[6] Ikeda M (2018). A genome-wide association study identifies two novel susceptibility loci and trans population polygenicity associated with bipolar disorder. Mol. Psychiatry.

[7] Nassir Ghaemi S (2005). Sensitivity and specificity of a new bipolar spectrum diagnostic scale. J. Affect. Disord..

[8] Aiken CB, Weisler RH, Sachs GS (2015). The Bipolarity Index: a clinician-rated measure of diagnostic confidence. J. Affect. Disord..

[9] Vazquez GH (2010). Screening for bipolar disorders in Spanish-speaking populations: sensitivity and specificity of the Bipolar Spectrum Diagnostic Scale-Spanish Version. Compr. Psychiatry.

[10] Zaratiegui RM (2011). Sensitivity and specificity of the mood disorder questionnaire and the bipolar spectrum diagnostic scale in Argentinean patients with mood disorders. J. Affect. Disord..

[11] Zimmerman M, Galione JN, Chelminski I, Young D, Ruggero CJ (2010). Performance of the Bipolar Spectrum Diagnostic Scale in psychiatric outpatients. Bipolar Disord..

[12] Hirschfeld RM (2000). Development and validation of a screening instrument for bipolar spectrum disorder: the Mood Disorder Questionnaire. Am. J. psychiatry.

[13] Egeland JA (1987). Bipolar affective disorders linked to DNA markers on chromosome 11. Nature.

[14] Hostetter AM, Egeland JA, Endicott J (1983). Amish Study, II: consensus diagnoses and reliability results. Am. J. psychiatry.

[15] Strauss KA (2014). A population-based study of KCNH7 p.Arg394His and bipolar spectrum disorder. Hum. Mol. Genet..

[16] Beiler K. *Descendants and history of Christian Fisher*, 1757-1838. 4th edn. (Grand Rapids MI, HeuleGordon, 2009).

[17] Shirk L., Shirk B. *Directory of the Groffdale Conference Mennonite churches*. 5th edn. (L.N. & B.N. Shirk, Kutztown Pennsylvania, 2007).

[18] Mitchell BD (2012). Living the good life? Mortality and hospital utilization patterns in the Old Order Amish. PLoS ONE.

[19] Insel TR (2014). The NIMH Research Domain Criteria (RDoC) Project: precision medicine for psychiatry. Am. J. Psychiatry.

[20] Perkins NJ, Schisterman EF (2006). The inconsistency of “optimal” cutpoints obtained using two criteria based on the receiver operating characteristic curve. Am. J. Epidemiol..

[21] Kochunov P (2016). The common genetic influence over processing speed and white matter microstructure: evidence from the Old Order Amish and Human Connectome Projects. NeuroImage.

[22] Almasy L, Dyer TD, Blangero J (1997). Bivariate quantitative trait linkage analysis: pleiotropy versus co-incident linkages. Genet. Epidemiol..

[23] Williams-Blangero S, Blangero J (1992). Quantitative genetic analysis of skin reflectance: a multivariate approach. Hum. Biol..

[24] Hong LE (2006). Familial aggregation of eye-tracking endophenotypes in families of schizophrenic patients. Arch. Gen. Psychiatry.

[25] Green EK (2013). Replication of bipolar disorder susceptibility alleles and identification of two novel genome-wide significant associations in a new bipolar disorder case-control sample. Mol. Psychiatry.

[26] Purcell SM (2009). Common polygenic variation contributes to risk of schizophrenia and bipolar disorder. Nature.

[27] Contreras J, Hare E, Chavarria G, Raventos H (2018). Quantitative genetic analysis of anxiety trait in bipolar disorder. J. Affect. Disord..

[28] Fears SC (2014). Multisystem component phenotypes of bipolar disorder for genetic investigations of extended pedigrees. JAMA Psychiatry.

[29] Greenwood TA (2013). Heritability and linkage analysis of personality in bipolar disorder. J. Affect. Disord..

[30] Zimmerman M, Ruggero CJ, Chelminski I, Young D (2008). Is bipolar disorder overdiagnosed?. J. Clin. Psychiatry.

[31] Kelsoe JR (2003). Arguments for the genetic basis of the bipolar spectrum. J. Affect. Disord..

[32] Nusslock R, Frank E (2011). Subthreshold bipolarity: diagnostic issues and challenges. Bipolar Disord..

[33] Zimmermann P (2009). Heterogeneity of DSM-IV major depressive disorder as a consequence of subthreshold bipolarity. Arch. Gen. Psychiatry.

[34] Hoertel N, Le Strat Y, Angst J, Dubertret C (2013). Subthreshold bipolar disorder in a U.S. national representative sample: prevalence, correlates and perspectives for psychiatric nosography. J. Affect. Disord..

[35] Fiedorowicz JG (2011). Subthreshold hypomanic symptoms in progression from unipolar major depression to bipolar disorder. Am. J. Psychiatry.

[36] Gill KE, Cardenas SA, Kassem L, Schulze TG, McMahon FJ (2016). Symptom profiles and illness course among Anabaptist and Non-Anabaptist adults with major mood disorders. Int. J. Bipolar Disord..

[37] Strauss KA (2006). Recessive symptomatic focal epilepsy and mutant contactin-associated protein-like 2. N. Engl. J. Med..

[38] Arking DE (2008). A common genetic variant in the neurexin superfamily member CNTNAP2 increases familial risk of autism. Am. J. Hum. Genet..

[39] Rossi E (2008). A 12Mb deletion at 7q33-q35 associated with autism spectrum disorders and primary amenorrhea. Eur. J. Med. Genet..

[40] Albert JS (2014). Null mutation in hormone-sensitive lipase gene and risk of type 2 diabetes. N. Engl. J. Med..

[41] Pollin TI (2008). A null mutation in human APOC3 confers a favorable plasma lipid profile and apparent cardioprotection. Science.

[42] Ruderfer DM (2014). Polygenic dissection of diagnosis and clinical dimensions of bipolar disorder and schizophrenia. Mol. Psychiatry.

